# Downregulation of NCOA4 expression indicates poor prognosis and promotes the progression of cholangiocarcinoma

**DOI:** 10.1371/journal.pone.0327722

**Published:** 2025-08-11

**Authors:** Wenlong Shen, Yi Liu, Bing Dai, Changling Qin, Yongli Fu, Xi Li, Chi Liu

**Affiliations:** 1 The Department of Pancreatic Surgery, General Surgery Department, Nanyang City Center Hospital, Nanyang, Henan, China; 2 Life Science Research Center, The First Affiliated Hospital of Xinxiang Medical University, Xinxiang, China; 3 Department of Hand, Foot and Joint Surgery, Nanyang City Center Hospital, Nanyang, China; 4 Department of Scientific Research, Nanyang City Center Hospital, Nanyang, China; University of Pennsylvania, UNITED STATES OF AMERICA

## Abstract

The aim of this study was to investigate how the cholangiocarcinoma cell lines RBE and HCCC-9810 responded to NCOA4 downregulation in terms of proliferation, migration and invasive.First,we analyzed the differential expression and survival prognosis of the NCOA4 gene using a bioinformatic approach,as well as validation using clinical samples.Next,cholangiocarcinoma cells were cultured and the NCOA4 gene was down-regulated with siRNA,and then NCOA4 and GPX4 expression was detected using qPCR and Western blot.Cell was measured using CCK8, cell cloning, wound healing, and transwell migration and invasion.Levels of changes in indicators related to ferroptosis were measured after induction of iron metamorphosis by Erastin. Data from TCGA showed that NCOA4 shows greater downgrade in tumor tissues than in non-tumor tissues and the overall survival (OS) of patients with low NCOA4 expression was significantly shorter than that of patients with high NCOA4 expression.The qPCR results showed that NCOA4 was expressed at low levels in cholangiocarcinoma tissue specimens; the mRNA expression of NCOA4 decreased after knocking down NCOA4 in cells. Western blot (WB) analysis showed that NCOA4 downregulation led to an increase in GPX4 expression. The cell cloning assay confirmed that downregulation of NCOA4 significantly increased cell viability. The transwell and wound healing assays demonstrated that the proliferation rate increased after downregulation of NCOA4. After NCOA4 silencing, ferroptosis indicators such as Fe^2+^, MDA, and ROS expression were lowered;GSH expression was increased.Our findings indicated the regulatory effects of NCOA4 on GPX4 protein and its contribution to malignant progression in CCA, which could provide a potential therapeutic target for CCA.

## Introduction

Cholangiocarcinoma, a highly aggressive and malignant cancer, poses a significant threat to human life and health [1]. Comprising approximately 15% of all primary liver tumors and 3% of gastrointestinal tumors, it is a prevalent concern [2,3]. With early-stage metastasis and a poor prognosis, morbidity and mortality rates have been on the rise for the past 30 years [4]. Primary sclerosing cholangitis, congenital choledocholithiasis, hepatic bile duct stones and hepatic schistosomiasis are major risk factors for Cholangiocarcinoma [5]. Surgery is currently the only possible cure, but most patients are diagnosed at an advanced stage and lose the chance of surgery [6]. At this stage, chemotherapy is still the main treatment for advanced cholangiocarcinoma, and even with aggressive chemotherapy, there is still a high lethality rate and an overall survival of less than 12 months [7]. To date, only one targeted drug—the FGF12 receptor inhibitor—is selectively utilized in a limited subset of CCA cases. patients,still has some limitations [8]. Investigating the molecular underpinnings of Cholangiocarcinoma development and pinpointing predictive biomarkers and therapeutic targets are crucial for advancing clinical management of the disease.

Nuclear receptor coactivator 4 (NCOA4), earlier named androgen receptor-associated protein 70 (ARA70), is an extremely important key regulator of ferroptosis [9,10]. NCOA4 interacts with and is co-activated by multiple nuclear hormone receptors to maintain genomic stability [9]. NCOA4 is closely related to the development of many cancers, plays a regulatory role in their biological behavior, and is involved in tumor growth and metastatic spread [11]. Abnormal NCOA4 expression is observed in various tumor cells, and its abnormal expression can contribute to the malignancy of some tumor cells and is significantly associated with cancer prognosis [12]. In cancers such as cholangiocarcinoma, colon adenocarcinoma, and glioma, NCOA4 is expressed at low levels, and the overall survival of patients is reduced [13]. In lung adenocarcinoma, the silencing of NCOA4 gene expression significantly promotes tumor cell proliferation, invasion, and metastasis [14]. Huang and Qu et al. found [15,16] that iron-dependent cell death was inhibited and cell proliferation and metastasis were significantly increased after NCOA4 knockdown in human hepatocellular carcinoma HepG2 and human pancreatic cancer PANC1 cells. Recently, researchers have discovered that decreased levels of NCOA4 alleviate cerebral white matter damage resulting from subarachnoid hemorrhage and impede the progression of esophageal cancer [17]. Ferroptosis, a mode of cell death discovered relatively recently, occurs frequently during tumor development and s primarily characterized by the inactivation of glutathione peroxidase 4 (GPX4) [18]. Guerriero et al. showed [19] that high GPX4 expression in hepatocellular carcinoma promotes the proliferation and metastasis of cancer cells. The investigators speculated whether NCOA4 is involved in the development of cholangiocarcinoma through the mechanism underlying GPX4-mediated iron-dependent cell death.

The role and mechanism of action of NCOA4 in cholangiocarcinogenesis, metastasis, and infiltration are yet to be reported. In this study, we selected cholangiocarcinoma cells and used gene silencing to downregulate NCOA4 expression in cells to investigate its effect on the proliferation, metastasis, and invasive ability of cholangiocarcinoma cells and identify the potential mechanism of ferroptosis. We believe this will help identify potential targets for predicting the treatment and prognosis of cholangiocarcinoma.

## Materials and methods

### 1 Bioinformatics analysis

Gene expression profiling interaction analysis (GEPIA; gepa.oma-pku.cn/) was performed to analyze NCOA4 expression and overall survival (OS) in cancer tissues (n = 36) and paracancerous tissues (n = 9), including the relative risk ratio (HR) and degree of clinicopathological correlation. (Data accessed on April 20, 2024)

### 2 Clinical specimens

Cholangiocarcinoma tissues and paracarcinoma tissues (>2 cm from the cancerous tissues) were obtained from patients who underwent surgical resection from January to July 2022 at the First Affiliated Hospital of Xinxiang Medical College. Patients with cholangiocarcinoma were pathologically diagnosed by two specialists from The First Affiliated Hospital of Xinxiang Medical College. Patients included in this study underwent surgical therapy. No patient received treatment before their surgery. The tumors and matched normal tissues from the patients were excised and frozen. Subsequently, the tissue specimens were stored in freezers at −80 °C. 12 samples were obtained before clinical treatments (chemotherapy or radiotherapy). The study protocol for human experiments was approved by the ethics committee of the hospital (No.2020083). This study adhered to the standards set by the Declaration of Helsinki. All patients were informed and consented to the use of their specimens for clinical diagnosis, treatment, and scientific research.

### 3 Cell culture

Human CCA cell line RBE (CL-0191) was purchased from Wuhan Pronsai Life Sciences Co. Human CCA cell line HCCC-9810 (CC0117) was purchased from Guangzhou Saiku Biotechnology Co. Cells were cultured in RPMI-1640 medium supplemented with 10% fetal bovine serum (FBS). Cells were maintained in a 37 °C, 5% CO2 incubator, and the complete culture medium was refreshed every 2–3 days. BS was obtained from Gibco, and RPMI-1640 medium was obtained from Solarbio (Beijing, China).

### 4 Cell transfection

To temporarily silence the NCOA4 gene in RBE/HCCC-9810 cells, siRNA was transfected into the cells using Lipofectamine™ 2000 (Invitrogen) according to the manufacturer’s instructions. siRNA (i.e., siNCOA4−1, siNCOA4−2, and siNCOA4−3) and siRNA negative control (NC) were purchased from Genepharma (Shanghai, China);the sequences are shown in Table 1.The silencing efficiency was confirmed using RT-qPCR. The siRNA sequences with the highest silencing efficiency were selected, and cells were collected 24 h or 48 h after transfection for further experiments.

**Table 1:** siRNA sequences and negative controls sequences.

Name	Sequence number
Negative control	
forward	5’-UUC UCC GAA CGU GUC ACG UTT-3’
reverse	5’-ACG UGA CAC GUU CGG AGA ATT-3’
si-NCOA4–1	
forward	5’-GGG CUG AAC AGC AAA UUA ATT-3’
reverse	5’-UUA AUU UGC UGU UCA GCC CTT-3’
si-NCOA4–2	
forward	5’-GCA GCU UAA AGU UGA UAA ATT-3’
reverse	5’-UUU AUC AAC UUU AAG CUG CTT-3’
si-NCOA4–3	
forward	5’-CUG GCA AAC AGA AGU UUA ATT-3’
reverse	5’-UUA AAC UUC UGU UUG CCA GTT-3’

### 5 RNA extraction and real-time PCR

According to the manufacturer’s instructions, total RNA was extracted from the culture cells and tumor tissues using TRIZOL (Accurate Biology Co, HuNan, China). Reverse transcription was performed using the specimens (Invitrogen Co, USA). Amplification was achieved using the SYBR green master mix (Invitrogen Co). qPCR was performed on ExicyclerTM 96 (Bioneer Co., Korea); the primer sequences are shown in Table 2. The relative expression of each target mRNA in each sample was normalized to GAPDH expression. The qRT-PCR conditions were as follows: 95 °C for 1 min; 95 °C for 20 s, 60 °C for 1 min, 40 cycles. The 2-ΔΔCt method was used to calculate the relative expression level of NCOA4 mRNA.

**Table 2:** Primer sequences for RT-qPCR.

Name	Sequence number
NCOA4	
forward	5’-GCTTGCTATTGGTGGAGTTCTCC-3’
reverse	5’-GCCATACCTCACGGCTTCTAAG-3’
GAPDH	
forward	5’-CAGGAGGCATTGCTGATGAT-3’
reverse	5’-GAAGGCTGGGGCTCATTT-3’

NCOA4 (nuclear receptor coactivator 4).

GAPDH (Glyceraldehyde-3-phosphate dehydrogenase).

### 6 Western blot

Whole-cell lysates were prepared in RIPA buffer (Epizyme). The protein concentration of extracts was measured using a BCA protein assay kit (Beyotime, China). The protein samples were separated by SDS-PAGE and transferred to PVDF membranes. Next, TBST buffer containing 5% (M/V) non-fat dry milk was applied to block the membranes for 1 h. The blots were probed with appropriate primary antibodies overnight at 4 °C. After washing with TBST buffer, the membranes were treated with the corresponding secondary antibodies for 1 h at 37 °C, followed by ECL detection. Finally, we used the ImageJ software for relative protein analysis. The antibodies used are listed in Table 3.

**Table 3:** Antibodies used in the study.

Antibody	Applications	Supplier
Primary antibodies		
NCOA4	WB	Proteintech (39896)
GPX4	WB	Proteintech (67763–1-Ig)
HRP-conjugated secondary antibodies		
Goat Anti-Rabbit IgG-HRP	WB	Proteintech (SA00001–9)
Goat Anti-Mouse IgG-HRP	WB	Proteintech (SA00001–8)
Internal antibody		
GAPDH	WB	proteintech (10494–1-AP)

NCOA4 (nuclear receptor coactivator 4);

GPX4 (Glutathione peroxidase 4);

GAPDH (Glyceraldehyde-3-phosphate dehydrogenase).

### 7 Cell proliferation and colony formation assays

We used Cell Counting Kit-8 (CCK8, Solarbio, China) to assess cell proliferation. We transfected the cells with siRNA. After 24 h of transfection, the said cells were inoculated in 96-well plates (3 × 103 cells/well) and incubated at 37 °C with 5% CO2. After the first, second, third, fourth, and fifth days, CCK-8 reagent (10 μL/well) was added to each well. Later, the plates were incubated at 37 °C for 2 h. The optical density (OD) was measured at 450 nm using an 800TS microplate reader (BIOTEK Instruments, VT, USA). In the colony formation assay, we inoculated 50 cells into 6-well dishes and then treated them with siRNA. After transfection for 24 h, the old medium was aspirated, new medium was added, and the cells were incubated in an incubator for 2 weeks. After rinsing with PBS, the colonies were fixed with 4%paraformaldehyde and then stained with crystal violet solution (Solarbio, China) for 15 min. The number of clones formed in each group of cells was calculated using ImageJ software.

### 8 Transwell assay

The transiently transfected cells were harvested and suspended in a serum-free medium. The transwell assay was performed to analyze the cell migration and invasion potential. Briefly, 100 μL(Contains 1x10^5^ Cells)of cell suspension was added into the upper chambers of the 24-well Transwell (1 × 100). Subsequently, 600 μL of medium containing 10% FBS was added to the lower chambers.The upper chambers were coated with Matrigel (Biosciences, USA) for the cell invasion assays. After incubation for 24 h at 37 °C and 5% CO2 under humidity-saturated conditions, the Transwell chambers were washed with PBS, fixed in 4% paraformaldehyde, and stained in crystal violet solution (Solarbio, China). The migrated and invading cells were quantified under a microscope (IX53, OLYMPUS, Japan).

### 9 Wound healing assays

We cultured the cells in 6-well plates. The cells were scratched using a 200-µL sterile pipette tip, then washed three times with PBS, and subsequently cultured for 48 hours. The medium utilized did not contain FBS.Images were taken at 0, 24, and 48 h after focusing on the same position. The ImageJ software was used to calculate the migration rate in each group of cells.Cell migration rate = (initial scratch width – scratch width after 48 hours)/initial scratch width x 100%.

### 10 Detection of levels of key indicators of ferroptosis

After treatment of CCA cells with the ferroptosis inducer Erastin, NCOA4 was knocked down and the DMSO-treated group was used as a control group. The content of Fe^2+^ in the cells was detected using the Ferrous Ion Content Assay Kit (Solarbio, China).The amount of MDA in the cells was detected using the Malondialdehyde (MDA) Assay Kit (Beyotime, China).The expression level of ROS in cells was detected using the Reactive Oxygen Species (ROS) Assay Kit (Jiancheng Bioengineering, China).The expression level of GSH in cells was detected using the Reduced Glutathione (GSH) Assay Kit (Jiancheng Bioengineering, China).

### 11 Statistical analysis

Statistical analysis was performed using GraphPad Prism 8, and images were created using PS. The data are presented as mean ± standard deviation (SD). Overall survival curves were plotted using the Kaplan-Meier method, and relative risk ratios (HR) were analyzed using the Mantel-Cox test. Differences between two groups were analyzed by the Student’s t-test. Values with P < 0.05 were considered statistically significant.

## Results

### 1 Low NCOA4 expression in human CCA tissues is correlated with poor prognosis

**Fig 1:**
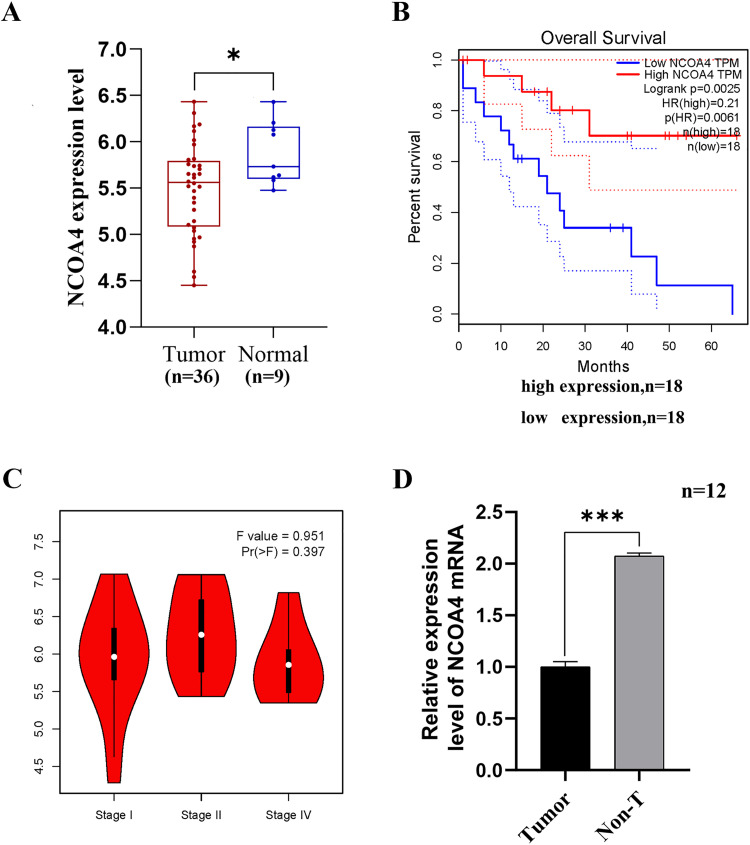
NCOA4 is downregulated in human CCA tissues and is correlated with poor prognosis. (A) The box plot was generated based on data from the GEPIA database (http://gepia.cancer-pku.cn/), cancer tissues (n = 36) and paracancerous tissues (n = 9), *P < 0.05. (B) The expression of NCOA4 was positively correlated with the overall survival of patients with cholangiocarcinoma; relative hazard ratio (HR)=0.21, Sample of two groups n = 18, both *P < 0.01. (C) Correlation between NCOA4 expression and the clinicopathological stage of patients. (D) The expression levels of NCOA4 in CCA tissues and paired non-cancerous tissues were determined using RT-qPCR, n = 12,***P < 0.001.All data are expressed as Mean ± SD.

To investigate the role of NCOA4 in CCA progression, we first analyzed the data from GEPIA, which is a useful web server for gene expression analysis based on data from tumors and normal samples from the TCGA and GTEx databases. The results revealed that NCOA4 expression was significantly lower in cholangiocarcinoma tumor samples compared to normal tissues (Fig 1A). Survival analysis demonstrated a positive correlation between NCOA4 expression and overall survival (OS) in a cohort of 18 patients, with a relative risk ratio (HR) = 0.21 (Fig 1B). The degree of correlation between NCOA4 expression and patient pathological stage showed no significant differences by stage (Fig 1C). We investigated the differential expression of NCOA4 in cholangiocarcinoma tissues and paired non-cancerous tissues and found that NCOA4 expression was significantly lower in cholangiocarcinoma tissues than in normal tissues (Fig 1D). These results implied that NCOA4 may be involved in CCA progression. To verify our hypothesis, we then silenced NCOA4 in two CCA cell lines (RBE and HCCC-9810) for the subsequent experimental studies.

### 2 Transfection efficiency of si-NCOA4 in CCA cells and associated protein expression levels after NCOA4 knockdown

**Fig 2:**
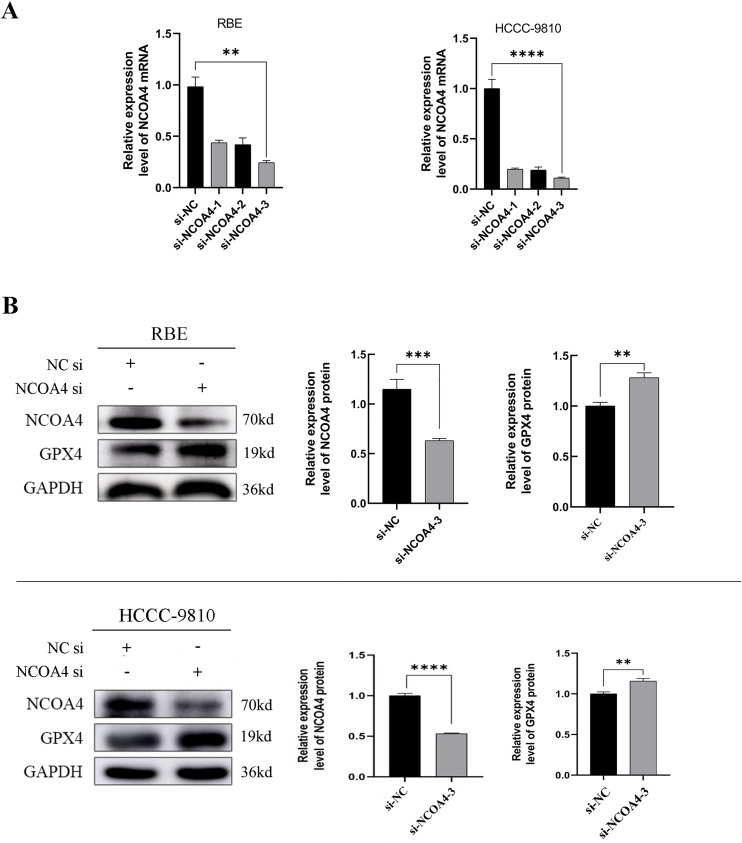
NCOA4 protein expression is downregulated and GPX4 protein expression was significantly upregulated in CCA cells. (A) The transfection efficiency of si-NCOA4 and their respective controls was detected using RT-qPCR in CCA cells. **P < 0.01,****P < 0.0001. (B) After down-regulation of NCOA4 expression using siRNA, Measurement of NCOA4 and GPX4 protein expression levels in RBE and HCC-9810 cells by western blot analysis. **P < 0.01,***P < 0.001 and****P < 0.0001.All data are expressed as Mean ± SD.

We successfully downregulated NCOA4 expression in RBE and HCCC-9810 cells transfected with NCOA4 siRNA (Fig 2A). Subsequently, in an in vitro study, we selected a highly efficient siRNA sequence for NCOA4 transfection. NCOA4 protein expression levels were decreased significantly in RBE and HCCC-9810 cells transfected with si-NCOA4–3 (Fig 2B). Compared with that in si-NC transfected cells, the GPX4 protein expression levels were significantly higher in si-NCOA4–3-transfected RBE and HCCC-9810 cells (Fig 2B). The results suggested that NCOA4 may promote CCA progression through the NCOA4/GPX4-mediated iron-mediated cell death signaling pathway.

### 3 Effect of siRNA transfection on the proliferation and clonogenicity of CCA cells

**Fig 3:**
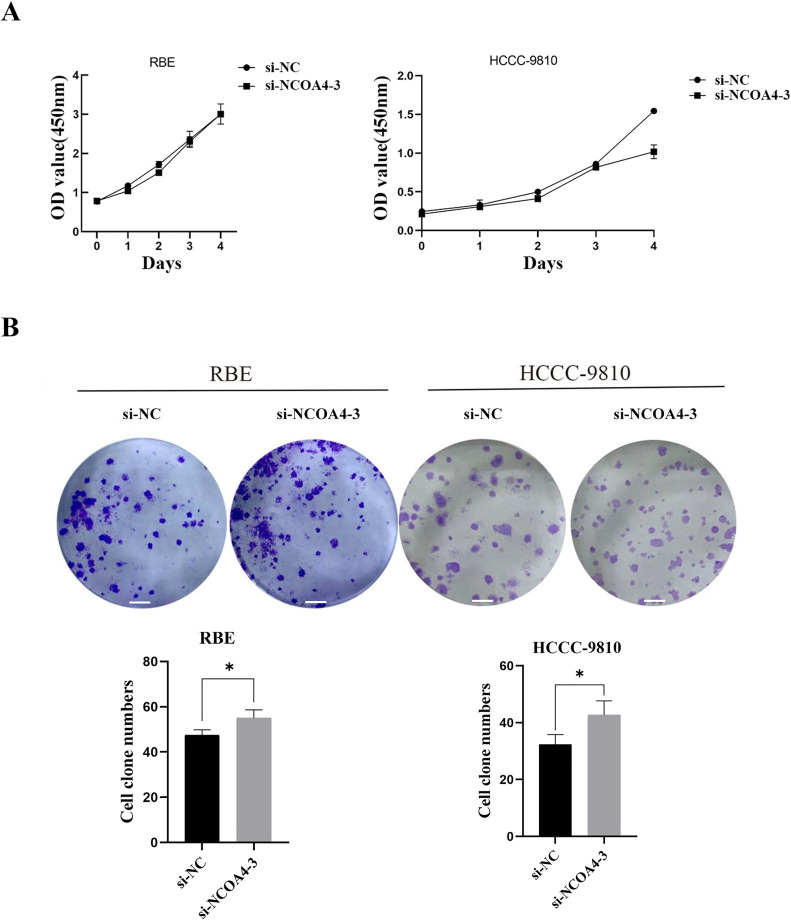
Low expression of NCOA4 promotes the proliferation of CCA cells in vitro. (A) CCK8 assays were used to assess the viability of NCOA4 knockdown in RBE and HCCC-9810 cells. (B) The proliferative cloning potential of RBE and HCCC-9810 cells after si-NCOA4 treatment was tested by plate cloning. Scale bars, 1000μm. *P < 0.05.All data are expressed as Mean ± SD.

To examine the role of NCOA4 in the progression of CCA, we used CCK8 assays to evaluate the effects of NCOA4 on the proliferation of RBE and HCCC-9810 cells. We observed no significant difference in cell proliferation in the si-NCOA4–3 group compared to that in the si-NC group (Fig 3A). The results of the plate cloning assay revealed that the number of visually observable cloned spheres formed by si-NCOA4–3 group cells increased significantly compared to that in si-NC in the negative control group (Fig 3B).

### 4 Effect of NCOA4 knockdown on CCA cell migration and invasion

**Fig 4:**
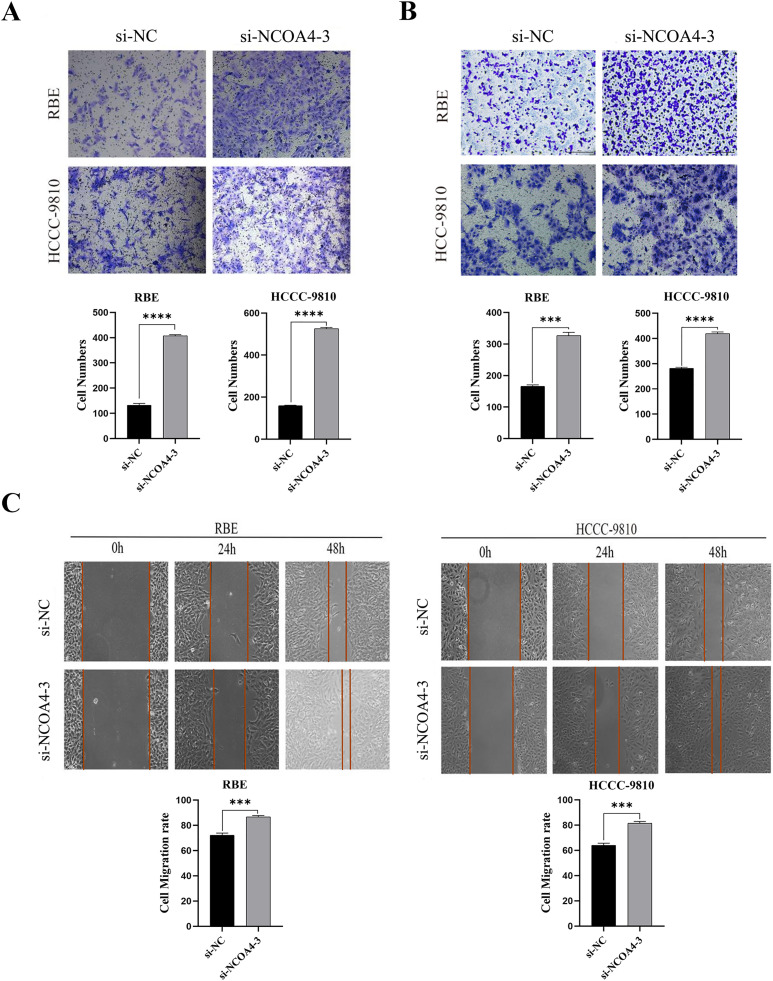
Low NCOA4 expression promotes the migration and invasion of CCA cells in vitro. (A and B) Migration and invasion analysis of RBE and HCC-9810 cells using transwell assay.Scale bars, 100μm. ***P < 0.001,****P < 0.0001. (C) The cell migration potential was measured using scratch-healing experiments.Scale bars, 100μm. ***P < 0.001.All data are expressed as Mean ± SD.

Migration and invasion are widely known as important signs of tumor metastasis. We assessed the effect of NCOA4 expression on cell migration and invasion. Transwell assays showed that NCOA4 knockdown promotes RBE and HCCC-9810 cell migration and invasion (Figs 4A and 4B). Similar results were observed in wound healing assays (Fig 4C). These findings suggest that the inhibition of NCOA4 promotes CCA cell development.

### 5 Effect of low NCOA4 expression after Erastin induction on the main indicators of ferroptosis in CCA cells in vitro

**Fig 5:**
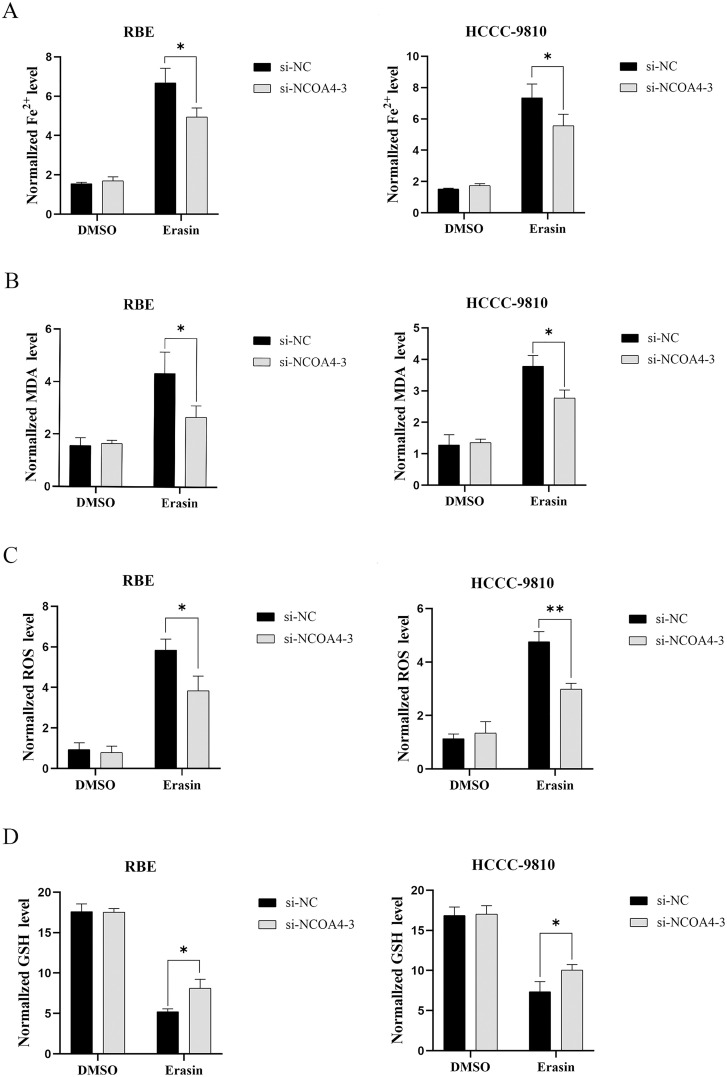
Low NCOA4 expression may inhibit ferroptosis in CCA cells in vitro. (A) Changes in iron ion levels in CCA cells. *P < 0.05. (B) Changes in MDA levels in CCA cells. *P < 0.05. (C) Changes in the level of ROS in CCA cells. *P < 0.05. (D)Changes in the level of GSH in CCA cells. *P < 0.05.All data are expressed as Mean ± SD.

We examined the main indicators of the ferroptosis process to verify whether NCOA4 could affect cholangiocarcinoma cell growth through the ferroptosis mechanism.After NCOA4 knockdown, Fe^2+^, MDA and ROS expression levels were reduced in RBE and HCCC-9810 cells (Fig 5A-5C).In addition, increased GSH expression levels (Fig 5D).These findings suggest that the inhibition of NCOA4 inhibits the onset of ferroptosis in CCA cells.

## Discussion

The incidence of cholangiocarcinoma has increased gradually in recent years. Moreover, its early diagnosis is challenging, it is prone to metastasis, and it has a poor prognosis. NCOA4 is a specific receptor that mediates the occurrence of autophagy in ferritin [20] and plays an important role in tumor development. NCOA4 has been shown to play a regulatory role in the malignant biological behavior of breast, prostate, pancreatic, and rectal cancers [21]. At present, the effect and mechanism of action of NCOA4 in the malignant biological behavior of cholangiocarcinoma cells are not clear.

The aggressiveness and metastasis of cholangiocarcinoma are critical factors affecting its treatment and prognosis. In particular, cell proliferation, invasion, and migration potential are key phenotypes that determine the metastatic potential of cholangiocarcinoma [22]. These factors contribute to the challenges in treating and predicting the outcome of cholangiocarcinoma, as evidenced by the poor prognosis and high mortality rates associated with the disease.The silencing of NCOA4 expression increased the migration and invasion of bile duct cancer cells and increased their clonogenic potential. In this study, after comparison with findings from related studies, we found that the regulation of NCOA4 expression in tumors is heterogeneous. High NCOA4 expression inhibits the proliferation of prostate cancer cells and promotes the proliferation and invasion of breast cancer cells [23]. Meanwhile, low expression of NCOA4 inhibits pancreatic cancer cell death [24]. The experimental results in this manuscript illustrate that NCOA4 is primarily involved in the invasion and migration of bile duct cancer cells, and the inhibition of NCOA4 expression promotes the migration and invasion potential of bile duct cancer cells. In addition, Yanhua Mou’s research indicates that patients with renal clear cell carcinoma exhibiting low NCOA4 expression have a reduced overall survival compared to those with high NCOA4 expression, aligning with the general trend that advanced stages of the disease are associated with poorer prognosis [25].

NCOA4 is a receptor with autophagic properties. It mediates iron-dependent autophagy and plays an important regulatory role in intracellular iron homeostasis [23]. Intracellular iron ion homeostasis relies on moderate iron-mediated autophagy. However, overactivation of autophagy can lead to excessive iron ion accumulation within cells, triggering ferroptosis, a process of iron-dependent cell death [26]. Iron-mediated cell death is a recently discovered mode of iron-dependent programmed cell death that occurs owing to lethal levels of lipid peroxidation [10]. NCOA4 mediates the phagocytosis of ferritin, a key regulator of iron-mediated cell death [27]. It plays a critical role in the process of iron-mediated cell death, and iron-mediated cell death is closely related to tumorigenesis and development and also regulates the invasive metastasis of malignant tumors [28]. Among the 12 clinical specimens we collected, cholangiocarcinoma tissues had significantly lower NCOA4 expression than paraneoplastic tissues. This suggests that a low expression of NCOA4 is likely to promote the malignant development of cholangiocarcinoma. In this study, the expression of NCOA4 and GPX4 in cholangiocarcinoma cells confirmed the presence of iron-mediated cell death in cholangiocarcinoma cells and its involvement in cellular regulation. Evidence shows that NCOA4 is required for iron-mediated cell death in certain cancer cell lines and MEF cells [29]. However, a major shortcoming of this study was that the collection of cholangiocarcinoma tissue specimens is challenging, and the sample size is considerably small to fully account for the differences in NCOA4 expression. We will continue to collect specimens and improve the supplementary experiments subsequently.

As glutathione peroxidase 4 (GPX4) is a hallmark protein in iron-mediated cell death, cellular susceptibility to iron-mediated death is decreased upon the enhancement of both GPX4 activity and expression, and vice versa [30]. Meanwhile, the lack of GPX4 expression as well as its increased depletion promotes iron-mediated cell death, which in turn promotes subsequent macrophage aggregation and activation, further promoting cell death [28]. NCOA4 can also regulate iron-mediated cell death. Low NCOA4 expression inhibits iron-mediated death, whereas high NCOA4 expression promotes iron-mediated cell death [31]. The carbonylation of HSP70 and α1-AT degrades GPX4 and promotes iron-mediated cell death, which leads to the progression and poor prognosis of cholangiocarcinoma [32]. We intended to explore whether NCOA4 can regulate iron-mediated cell death. We found that GPX4 expression increased as NCOA4 expression decreased after its knockdown. The experimental results of this study illustrate that NCOA4 knockdown increases GPX4 expression, inhibits iron-mediated cell death, and promotes the malignant behavior of cholangiocarcinoma cells. We found that iron autophagy-mediated cell death is closely related to liver fibrosis and neurodegenerative diseases and can be used to kill various cancer cells under specific circumstances [33]. The results of the study could see changes in the expression levels of ferroptosis-related indicators (e.g., Fe^2+^, MDA, ROS, GSH) in cholangiocarcinoma cells with low expression of NCOA4, which confirmed the presence of ferroptosis in cholangiocarcinoma cells and its involvement in cellular regulation. The above findings suggest that NCOA4 may influence the function of cholangiocarcinoma cells through iron-mediated cell death. The findings of this study, combined with data from the TCGA database, were used to perform bioinformatics analysis on the relationship between the metastasis and long-term survival of patients with cholangiocarcinoma. The 5-year prognostic survival rate of patients with cholangiocarcinoma with low NCOA4 expression was significantly lower than that of patients with high NCOA4 expression. This indicates that NCOA4 has a significant impact on the survival prognosis of patients with cholangiocarcinoma. Furthermore, Mining metabolic activity at single-cell resolution remains challenging. In the future, we can use scMetabolism [34] (http://www.cancerdiversity.asia/scMetabolism/) to analyze the relevance of NCOA4 to more cancer metabolism after collecting scRNA-seq Data, which we believe will be very interesting and novel.

In conclusion, the results of this study suggest that NCOA4 expression is closely related to the development of cholangiocarcinoma, and silencing NCOA4 expression can promote the proliferation, migration, and invasion of cholangiocarcinoma cells and is associated with the prognostic survival of patients with cholangiocarcinoma, and may play a role by reducing ferroptosis from occurring thus.

## Conclusion

The findings showed that NCOA4 downregual promoted cell proliferation,migration and invasive in RBE and HCCC-9810 via inhibiting ferroptosis,proving that NCOA4 is a novel gene linked with CCA.These outcomes imply the regulatory effects of NCOA4 on GPX4 protein and its contribution to malignant progression in CCA, which could provide a potential therapeutic target for CCA.

## Supporting information

S1 Raw images Western blotting images. (PDF)

S2 Language Certificate Language Certificate. (PDF)

S3 Tables siRNA, Primer sequences and Antibodies. (DOCX)
