# Supplementary material for: Downregulation of NCOA4 expression indicates poor prognosis and promotes the progression of cholangiocarcinoma
Source: PLoS One. 2025 Aug 11;20(8):e0327722. doi: 10.1371/journal.pone.0327722 (PMC12338789; doi:10.1371/journal.pone.0327722)
Supplement: S1 Raw images — (PDF) [file pone.0327722.s001.pdf]

Results in the manuscript (Figure 2 B): the format of the picture information is cell name + protein name + number. We used the NC group and experimental group results in the manuscript after the first blank group results without placing the first one.

RBE cell

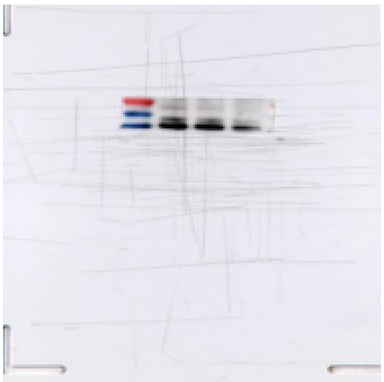

No.1

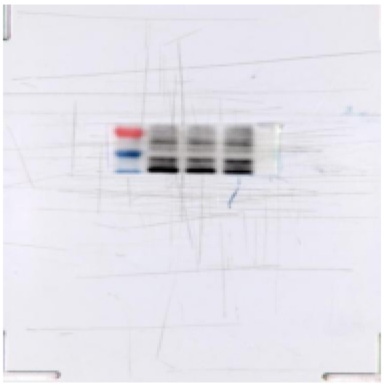

No.2

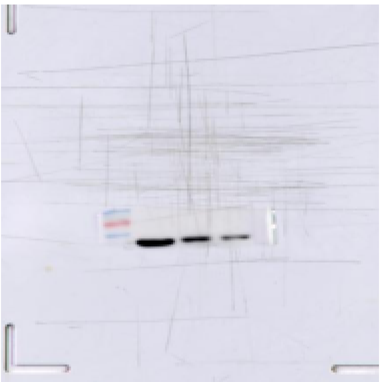

No.3

NCOA4

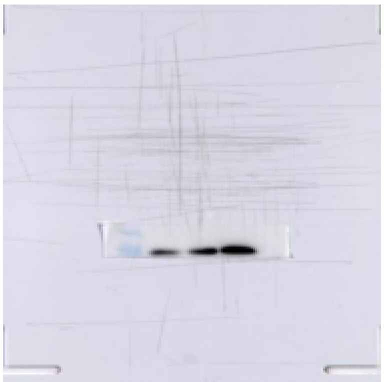

No.1

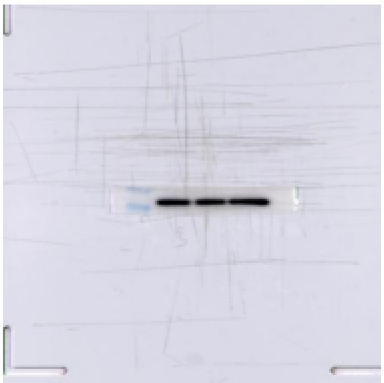

No.2

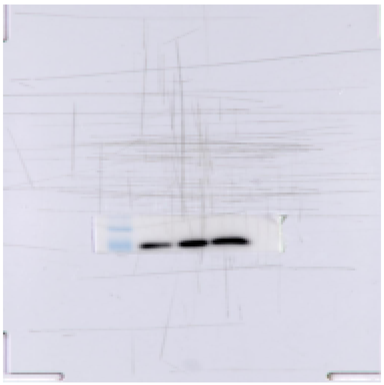

No.3

GPX4

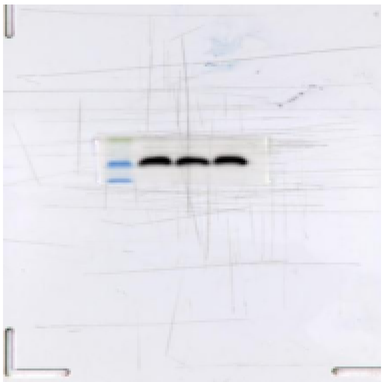

No.1

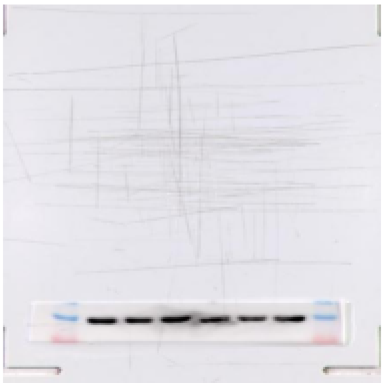

No.2

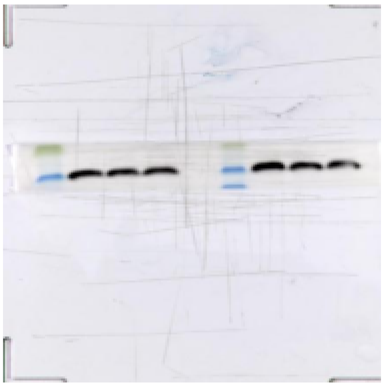

No.3

GAPDH

HCCC-9810 cell

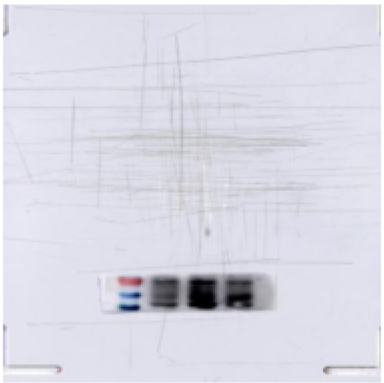

No.1

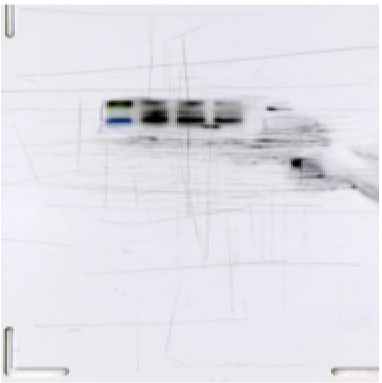

No.2

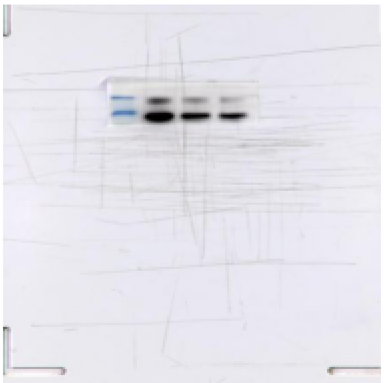

No.3

NCOA4

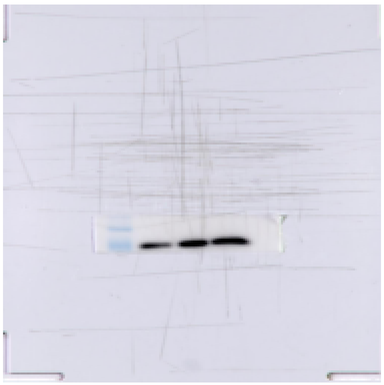

No.1

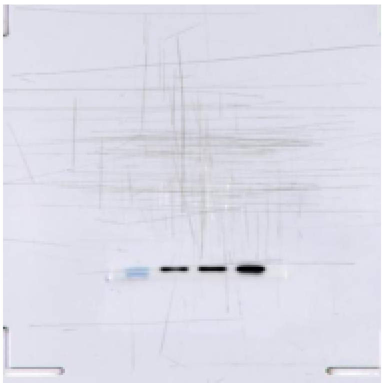

No.2

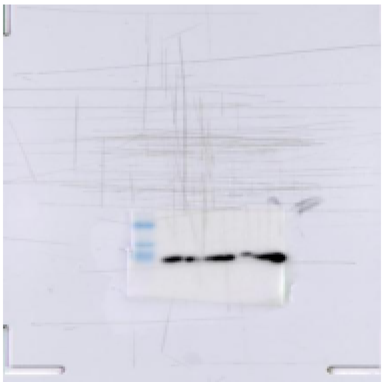

No.3

GPX4

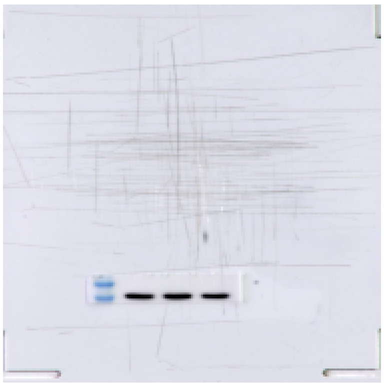

No.1

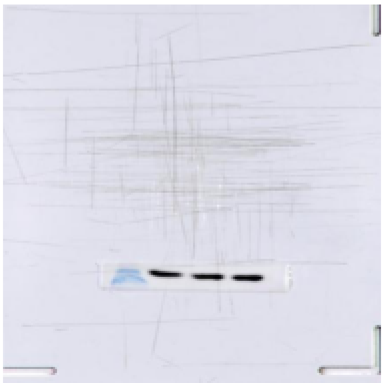

No.2

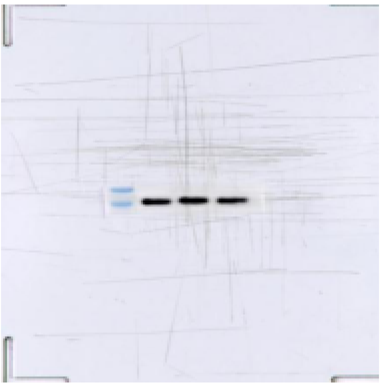

No.3

GAPDH
