# Supplementary material for: Downregulation of NCOA4 expression indicates poor prognosis and promotes the progression of cholangiocarcinoma
Source: PLoS One. 2025 Aug 11;20(8):e0327722. doi: 10.1371/journal.pone.0327722 (PMC12338789; doi:10.1371/journal.pone.0327722)
Supplement: S2 Language Certificate — (PDF) [file pone.0327722.s002.pdf]

Bullet Edits

EDITORIAL CERTIFICATE

This document certifies that the paper listed below was edited and proofread for proper English language, grammar, punctuation, spelling, and overall style by one or more than one highly qualified native speakers at Bullet Edits. All of the suggested amendments were tracked with the Microsoft Word "Track Changes" feature. Therefore, the author had the option to reject or accept each change individually.

Manuscript Title:

Downregulation of NCOA4 Expression Indicates Poor Prognosis and Promotes the Progression of Cholangiocarcinoma

Anti-counterfeiting Code:

3696fe42595720866cb7bdfd9714e698

Date Issued:

2023-02-19 16:37:31

Bullet Edits is a registered company headquartered in the UK with a global presence. We offer a range of editing, proofreading services to authors. Our Ph.D. editors are all native English speakers from the USA and UK. Authors who work with Bullet Edits are guaranteed excellent language quality and timely delivery.

BULLET EDITS LIMITED ★ COMPANY NO. 13345261 SEAL ★ ENGLAND AND WALES

Address: BULLET EDITS LIMITED, 85 Great Portland Street, London,UK
Tel: UK. (+44)20457 70286 / US.(+1)312-313-9179
Web: www.bulletedits.cn/Email: info@bulletedits.cn
VAT: GB 378 9316 43
