# Supplementary material for: Downregulation of NCOA4 expression indicates poor prognosis and promotes the progression of cholangiocarcinoma
Source: PLoS One. 2025 Aug 11;20(8):e0327722. doi: 10.1371/journal.pone.0327722 (PMC12338789; doi:10.1371/journal.pone.0327722)
Supplement: S3 Tables — (DOCX) [file pone.0327722.s003.docx]

**Table 1 siRNA sequences and negative controls sequences**

| **Name** | **Sequence number** |
| --- | --- |
| **Negative control** |  |
| forward | 5’-UUC UCC GAA CGU GUC ACG UTT-3’ |
| reverse | 5’-ACG UGA CAC GUU CGG AGA ATT-3’ |
| **si-NCOA4-1** |  |
| forward | 5’-GGG CUG AAC AGC AAA UUA ATT-3’ |
| reverse | 5’-UUA AUU UGC UGU UCA GCC CTT-3’ |
| **si-NCOA4-2** |  |
| forward | 5’-GCA GCU UAA AGU UGA UAA ATT-3’ |
| reverse | 5’-UUU AUC AAC UUU AAG CUG CTT-3’ |
| **si-NCOA4-3** |  |
| forward | 5’-CUG GCA AAC AGA AGU UUA ATT-3’ |
| reverse | 5’-UUA AAC UUC UGU UUG CCA GTT-3’ |

**Table 2** Primer sequences for RT-qPCR

| **Name** | **Sequence number** |
| --- | --- |
| **NCOA4** |  |
| forward | 5’-GCTTGCTATTGGTGGAGTTCTCC-3’ |
| reverse | 5’-GCCATACCTCACGGCTTCTAAG-3’ |
| **GAPDH** |  |
| forward | 5’-CAGGAGGCATTGCTGATGAT-3’ |
| reverse | 5’-GAAGGCTGGGGCTCATTT-3’ |

NCOA4 (nuclear receptor coactivator 4)；

GAPDH (Glyceraldehyde-3-phosphate dehydrogenase)

**Table 3** Antibodies used in the study

| **Antibody** | **Applications** | **Supplier** |
| --- | --- | --- |
| **Primary antibodies** |  |  |
| NCOA4 | WB | Proteintech (39896) |
| GPX4 | WB | Proteintech (67763-1-Ig) |
| **HRP-conjugated secondary antibodies** |  |  |
| Goat Anti-Rabbit IgG-HRP | WB | Proteintech (SA00001-9) |
| Goat Anti-Mouse IgG-HRP | WB | Proteintech (SA00001-8) |
| **Internal antibody** |  |  |
| GAPDH | WB | proteintech (10494-1-AP) |

NCOA4 (nuclear receptor coactivator 4)；

GPX4 (Glutathione peroxidase 4);

GAPDH (Glyceraldehyde-3-phosphate dehydrogenase);
